# Using genomic signatures for HIV-1 sub-typing

**DOI:** 10.1186/1471-2105-11-S1-S26

**Published:** 2010-01-18

**Authors:** Aridaman Pandit, Somdatta Sinha

**Affiliations:** 1 Centre for Cellular and Molecular Biology (CSIR), Hyderabad, India

## Abstract

**Background:**

Human Immunodeficiency Virus type 1 (HIV-1), the causative agent of Acquired Immune Deficiency Syndrome (AIDS), exhibits very high genetic diversity with different variants or subtypes prevalent in different parts of the world. Proper classification of the HIV-1 subtypes, displaying differential infectivity, plays a major role in monitoring the epidemic and is also a critical component for effective treatment strategy. The existing methods to classify HIV-1 sequence subtypes, based on phylogenetic analysis focusing only on specific genes/regions, have shown inconsistencies as they lack the capability to analyse whole genome variations. Several isolates are left unclassified due to unresolved sub-typing. It is apparent that classification of subtypes based on complete genome sequences, rather than sub-genomic regions, is a more robust and comprehensive approach to address genome-wide heterogeneity. However, no simple methodology exists that directly computes HIV-1 subtype from the complete genome sequence.

**Results:**

We use Chaos Game Representation (CGR) as an approach to identify the distinctive genomic signature associated with the DNA sequence organisation in different HIV-1 subtypes. We first analysed the effect of nucleotide word lengths (k = 2 to 8) on whole genomes of the HIV-1 M group sequences, and found the optimum word length of k = 6, that could classify HIV-1 subtypes based on a Test sequence set. Using the optimised word length, we then showed accurate classification of the HIV-1 subtypes from both the Reference Set sequences and from all available sequences in the database. Finally, we applied the approach to cluster the five unclassified HIV-1 sequences from Africa and Europe, and predict their possible subtypes.

**Conclusion:**

We propose a genomic signature-based approach, using CGR with suitable word length optimisation, which can be applied to classify intra-species variations, and apply it to the complex problem of HIV-1 subtype classification. We demonstrate that CGR is a simple and computationally less intensive method that not only accurately segregates the HIV-1 subtype and sub-subtypes, but also aid in the classification of the unclassified sequences. We hope that it will be useful in subtype annotation of the newly sequenced HIV-1 genomes.

## Background

Human Immunodeficiency Virus (HIV) type 1, a retrovirus, is the causative agent of Acquired Immunodeficiency Syndrome (AIDS). With more than 33 million individuals living with the virus and more than 25 million deaths since its onset, HIV has led to a global pandemic [1]. The major problem to curb HIV-1, through the development of a vaccine, has been its high genetic variability and evolutionary rates [2]. This genetic heterogeneity of HIV-1 has been attributed to the lack of proofreading capabilities of the Reverse Transcriptase enzyme [3,4]. Genetically diverse population of viral species ('quasispecies') dwells inside an infected individual [4], and HIV can exhibit up to 10% variability within a single individual [5]. The human host's immune system as well as the antiviral drugs used in treatment regimes also trigger viral evolution.

Analogous to within-individual variability, HIV exhibits high heterogeneity at the population level. HIV-1 sequences are classified into three phylogenetically distinct groups - M (*Major*), O (*Outlier*), and N (*non-M/non-O*) - based upon their sequence diversity. The M group is globally prevalent and responsible for the pandemic. Group M is further stratified into nine genetically discrete subtypes - A to D, F to H, J, and K - showing up to 25% to 35% sequence level variations between the genomes in different subtypes [2,5,6]. The subtypes A and F are further classified into sub-subtypes (A1, A2) and (F1, F2) based upon differential clustering [2]. To add to the complexity, two or more HIV-1 subtypes recombine and circulate in the population to form Circulating Recombinant Forms (CRFs), and new CRFs continuously emerge over time.

Historically, the subtypes were classified based on the envelope (*env*) gene sequence variations and classification of subtypes A to F was done on the basis of *env *gene alone [7]. All subtypes, except E, could be consistently classified from the *gag *region of HIV-1 [8]. The partial genome sequences and phylogenies based on *env *and *gag *genes further led to designation of subtypes G to J [9-11]. Phylogenetic comparisons of A and F led to determination of sub-subtypes that form differential clusters within the corresponding subtypes [12]. Generally, HIV-1 strains fall into the appropriate phylogenetic clusters when multiple regions of their genome are analysed. Subtype K, which was earlier proposed to be a sub-subtype of F based upon phylogenetic analysis of *env *and *gag *sequences, was later classified as a distinct subtype when whole genome sequences were analysed [12]. Subtype I previously classified on the basis of C2V3 region of *env *sequences was later found to be a subtype A and G recombinant [10,13]. Some of the recent methods use *env*, *gag *and *pol *gene sequences, which together span most of the HIV genome. These studies clearly indicate that classification of subtypes based on complete genome sequences, rather than sub-genomic regions, may be a more robust and comprehensive approach. However, no simple methodology exists that directly computes a HIV-1 subtype from the complete genome sequence, rather than generating gene-based phylogenies and then analysing the distance matrices [14-16].

In this article, we address this problem by identifying the variations in the genomic signatures (at various word lengths) at whole genome level in the different subtypes of HIV-1 using the *Chaos Game Representation (CGR) *method[17]. CGR is a two-dimensional plot, where the primary sequence organisation of DNA is mapped using iterative functions. The use of CGR has mostly been restricted to a visualization tool representing nucleotide sequences, in which patterns like over- or under-representation of nucleotides, dinucleotides, trinucleotides etc. can be visually ascribed. Goldman concluded that the patterns exhibited by CGR are sufficient to evaluate word length composition of three, i.e., the frequencies of nucleotides, dinucleotides and trinucleotides [18]. However, it was shown later that longer oligonucleotide frequencies also influence the patterns seen in CGR [19]. Recently, a spectrum of word lengths, in addition to nucleotide and dinucleotide, in CGRs were identified as factors that can differentiate between genomes of different species. Several distance measures were proposed to compare two or more CGRs and it was employed for studying phylogenetic relationships among diverse species [19,20]. However, it is not clear if intra-species genomic variability, which is much less than between-species variation, can be resolved using CGRs with similar word lengths. A different class of methods, using data structures such as, *suffix arrays *and *suffix tree*, have also been used to study specific genomic signatures using different word lengths [21].

In this study, we demonstrate the applicability of CGR to address the problem of intra-species variability by considering the complex issue of HIV-1 subtype classification, as these subtypes form a set, which exhibit subtle differences that are sufficient for displaying differential infectivity and evolutionary dynamics [22]. We show that CGR is an effective methodology to resolve HIV-1 subtype variations by first optimising the suitable word length, and then applying the method to obtain the known and unknown HIV-1 subtypes by analysing all available whole genome sequences, along with the Reference Sequence set that is used by workers in the field [23]. Our studies clearly show that this unusual approach can effectively be used for studying intra-species variability in general, and specifically offer an easy-to-use and accurate method for HIV-1 sub-typing from whole genome data.

## Methods

### Data acquisition

HIV-1 sequences were downloaded in FASTA format from the HIV Sequence Database at Los Alamos National Laboratory (July 2009) [24]. The word length analysis was carried out on the dataset containing one sequence for each year corresponding to a subtype. This resulted in a set of 75 genome sequences containing 7 of subtype A, 26 of subtype B, 21 of subtype C, 13 of subtype D, and 8 of subtype G sequences. For word length optimisation, all subtype A and G sequences from the previous set were taken along with randomly choosing 8 each for subtype B, C and D. Thus, 39 genomes were used as the Training Set (Table 1), and the remaining sequences were used to test the optimised word length.

**Table 1:** Accession numbers of training set sequences used for word length optimisation

**Sr. No**.	A	B	C	D	G
**1**	AY521630	AF004394	U46016	AY773338	U88826
**2**	AM000053	AF042101	AY713415	AY773341	AF061642
**3**	AM000054	AF256204	AY713416	EF633445	AY772535
**4**	AY521629	AF086817	AY255826	AY322189	AY586549
**5**	AY521631	AF042103	EF514713	AF484516	AF423760
**6**	AM000055	AB428555	DQ207941	AJ488927	AY371121
**7**	DQ396400	AB287363	DQ369994	AY371157	AB231893
**8**		EU786678	EU786673	AY795907	EU786670

The Reference Sequences set was taken from the HIV Database, which were classified using traditional sequence alignment methods [23]. The Reference set contains four sequences each for subtype B, C, D and G; three sequence for subtype H; two each for subtype J and K; four each for sub-subtype A1, F1 and F2; two for sub-subtype A2 and four for SIVcpz, where SIVcpz is the SIV sequence derived from Chimpanzee. The U (unclassified) sequences were also collected from the database.

### CGR plot

CGR of a genome is plotted in a square, with each of the four vertices labelled as the four nucleotide bases A, T, G and C, respectively. To initialise, we place the first point in the middle of the square. The second point is placed as a mid-point between the initial point and the coordinates of the vertex corresponding to the first nucleotide of the DNA sequence. The next point, corresponding to the second nucleotide, is placed as a mid-point between the previously plotted point and the coordinate of the vertex. The process is repeated for the complete sequence and the entire genome is plotted in a two-dimensional plot. The frequency of different word lengths can be extracted by dividing the CGR space with a grid of appropriate size. To obtain the frequencies of all the k-letter words, CGR must be divided into a (2^k ^× 2^k^) grid. The frequencies are obtained by counting the number of occurrences in each box of the grid.

For our work, a (800 × 800) square was constructed to map the genomes. The four nucleotides were assigned CGR vertices as A (0,0); T (800,0); G (800,800); and C (0,800). For a genome *g* of length *n*, the position for nucleotide *g*_*i *_in the CGR is calculated as

where the initial point CGR_0 _is the mid point of the square (i.e., 400,400) and *i *varies from 1, .... n. In order to have greater resolution to study the effect of different word lengths, we have taken 800 divisions to construct the CGR.

**Figure 1:**
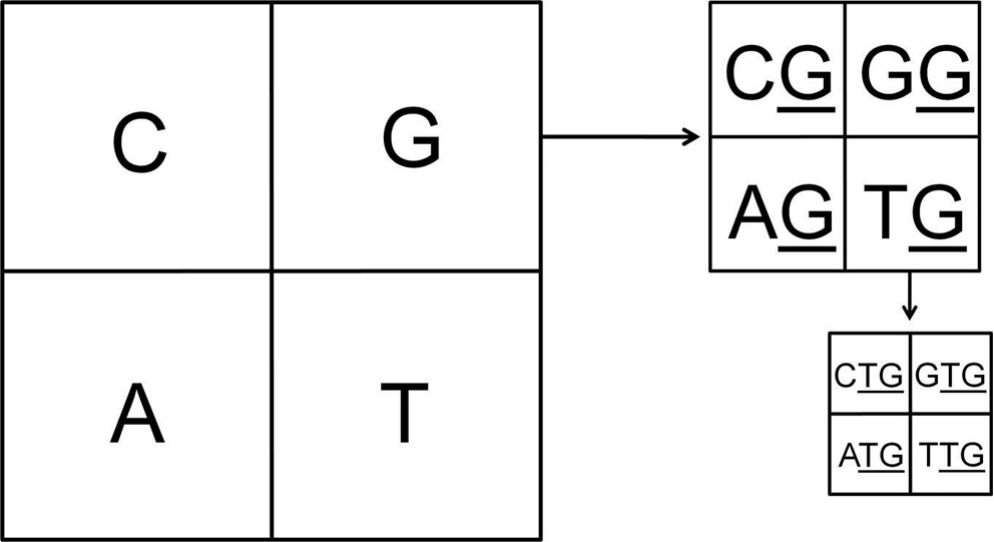
**Schematic explaining Chaos Game Representation**. The division of CGR to obtain dinucleotide and trinucleotide frequencies with the G quadrant shown sub-divided into the corresponding G-ending dinucleotides. The first letter is arranged following the pattern of single nucleotide used to plot the CGR, with G, C, A and T given from first to fourth quadrant respectively.

While calculating the dinucleotide frequencies, each of the quadrants corresponding to a nucleotide is divided into four parts. The first nucleotide of each part is then labelled as for the original CGR quadrant, while the second nucleotide is labelled as the quadrant for which the divisions are being made. The process is explained in Figure 1, where G quadrant is divided into the corresponding G ending dinucleotides, which can further be divided into corresponding trinucleotides, as shown for TG. Thus, for word length 2, the CGR is divided into 16 divisions, and inside each division, the frequency of occurrence is calculated. Similar iterative procedure is followed for higher word lengths. We have analysed the CGR up to word length k = 8.

### Distance calculations

To evaluate the relatedness of different HIV-1 subtypes, we calculate pair-wise distance between CGRs. We used simplest of the distances, the Euclidean distance *d*, between a pair of CGRs (A and B) using the formula:

where, k is the word length; a_ij _and b_ij _are the frequency values corresponding to the first and second CGR. For a given set of CGR, we constructed pair-wise distance matrices for each pair, and used it for further analysis. All calculations were performed using MATLAB R2007b [25].

### Clustering

Pair-wise distance matrices were used to cluster different HIV-1 subtypes using Neighbor-Joining (NJ) method and the "Neighbor" programme of PHYLIP [26] was used to construct the dendrograms.

## Results and discussion

Here we present the results of our study on the classification of HIV-1 subtypes using the CGR approach. First, we generated the CGR plots for the first HIV-1 complete genome sequence to highlight the features exhibited by a typical HIV-1 genome. Then we used the training set of HIV-1 subtype genome sequences, given in Table 1, to optimise the word length required to correctly segregate the different subtypes. We further tested the optimised word length on the Reference Sequence Set used for HIV sub-typing, and also for other subtype sequences available in the database. Finally, we analysed all the unclassified sequences for HIV-1 implementing our methodology of CGR.

### CGR for HIV genome

**Figure 2:**
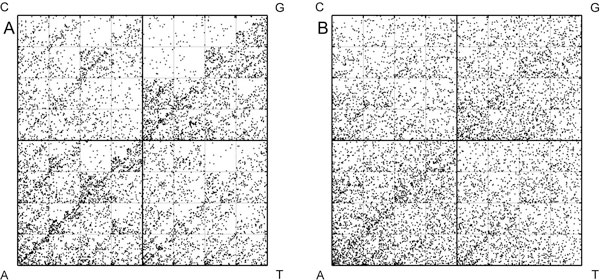
**Chaos Game Representation plots**. (A) HXB2, the first HIV-1 genome sequenced, and (B) random control of HXB2.

The first complete HIV-1 genome sequence (HXB2 - Accession number K03455) was taken to generate the corresponding CGR (Figure 2A). HIV-1 is a A-rich virus, which can be easily seen from the high density of points in the A-quadrant of the CGR. General properties like CG, CGG and CGT under-representation in this genome can be determined directly by looking at this CGR. As a control, we generated a randomised CGR keeping the single nucleotide composition as in HXB2 genome (Figure 2B). Given the constraint of single nucleotide composition, the random CGR comprises homogeneous frequency distribution. This random control also does not depict under-representation of CG, CGG and CGT dinucleotides. Clearly, CGR constructed for HIV-1 sequence highlights the prominent genome signatures.

### Clustering of HIV-1 group M subtypes using different word lengths

**Figure 3:**
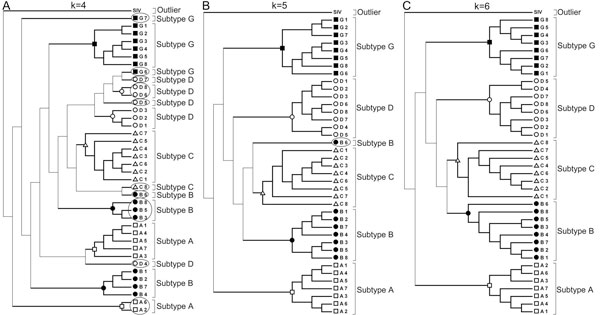
**Clustering of HIV-1 subtypes using different word lengths**. (A) k = 4, (B) k = 5 and (C) k = 6. SIV_CPZ _sequence was used as out-group to root the tree. The circled entries show the inaccurate clustering.

The Training Set of HIV-1 subtype sequences used to find the optimum word length sufficient to resolve various subtypes is given in Table 1. CGR corresponding to all Training Set genomes were constructed and the frequencies corresponding to word lengths k = 2 to 8 were calculated. Dendrograms constructed using the Neighbor-Joining algorithm for word lengths 4, 5 and 6 are shown in Figure 3. The SIV_CPZ _sequence is used as an out-group to root the Tree. Out of the 39 sequences, word lengths of 2 and 3 produced Trees with incorrect clustering of HIV-1 group M subtypes for 28 and 22 sequences, respectively. Word length of 4 generated a mixed cluster where 14 sequences do not cluster along with their respective subtypes (circled in Figure 3A). Here the cluster containing the maximum sequences for a particular subtype was assigned the "main" subtype cluster, and the rest of the sequences for that subtype were counted as being "incorrectly clustered". With word length 5, one sequence belonging to subtype B exhibit incorrect clustering with the subtype C cluster (Figure 3B). Accurate clustering was achieved with word length k = 6, where all Training Set subtypes segregated in distinct clusters. Similar clustering patterns were observed for subtypes with word length 7 and 8 (results not shown). Thus, increasing the word length did not provide any additional information. Hence, based on the Training Set sequences and the knowledge of existing subtype specifications, we chose word length k = 6 to be the optimum, as it can distinguish between HIV-1 subtypes with minimum computational cost.

### Resolving subtypes from the reference dataset

**Figure 4:**
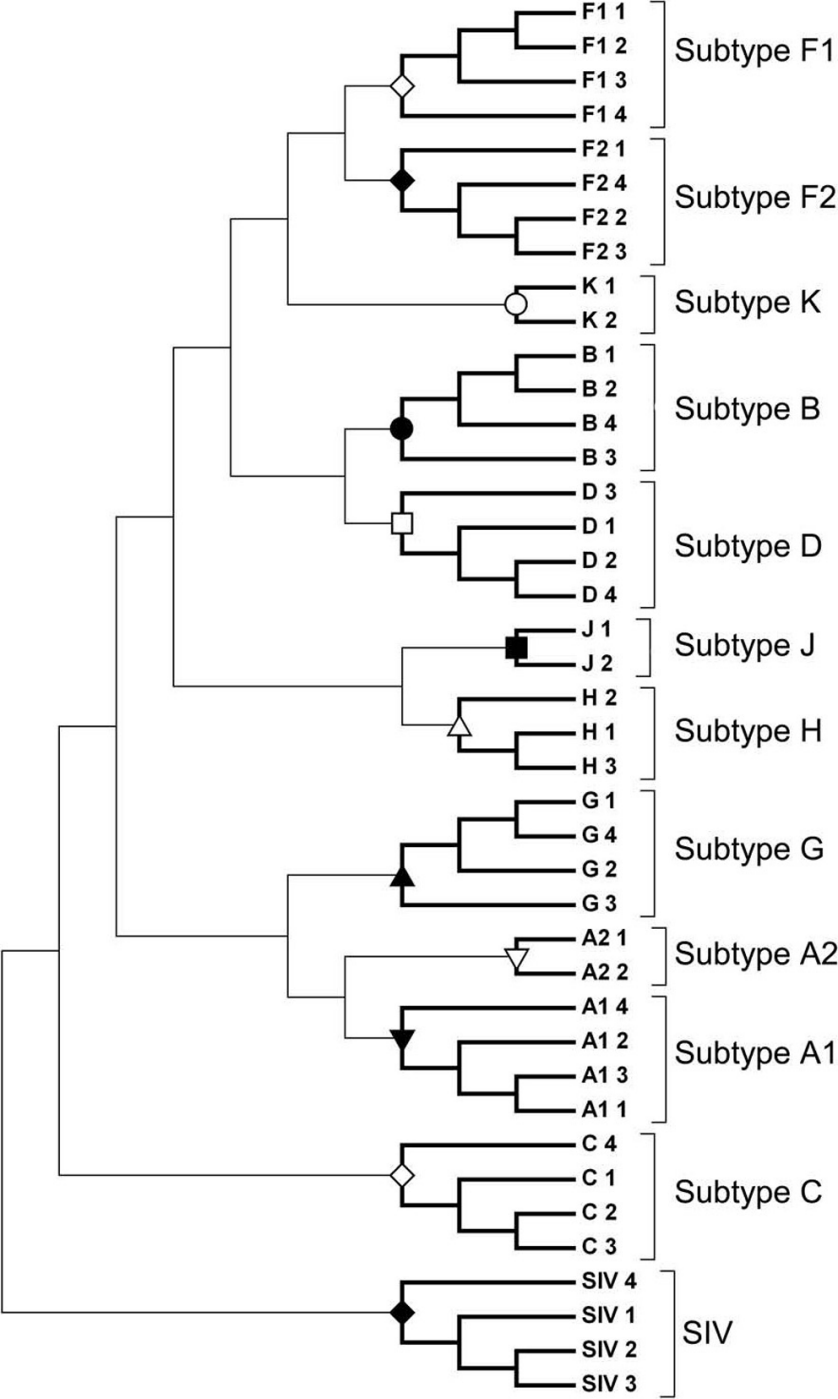
**Clustering for HIV-1 subtype reference sequence set for k = 6**. SIV was used as an out-group to root the tree.

In order to classify new HIV-1 sequences, a Reference Sequence Set, apart from the above-mentioned sequences, is often used [23]. CGR corresponding to all Reference Set genomes were constructed and the frequencies corresponding to word length 6 were calculated. Figure 4 shows the dendrogram of the Reference Set of HIV-1 group M sequences. It clearly resolves all the distinct clusters as per different subtypes given in the Reference Set [23]. Thus, the optimised word length generates consistent classification with the Reference Set sequences, which were classified using traditional sequence alignment methods. The data set was found to be free of any reticulate component by verifying it using the neighbor-net algorithm as implemented by SplitsTree4 [27].

### Resolving subtypes from all available genome sequences

**Figure 5:**
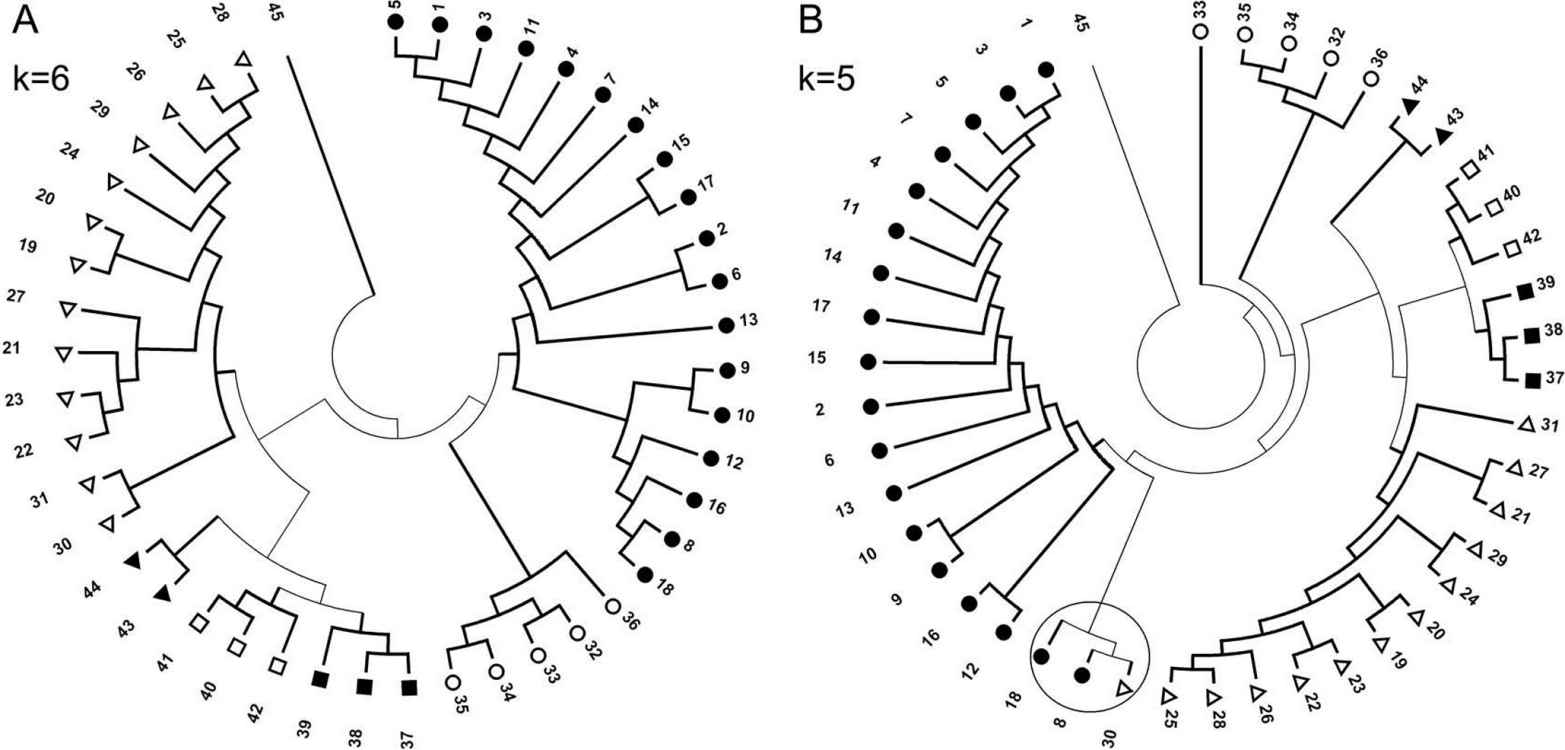
**Clustering of HIV-1 subtypes**. Clustering of HIV-1 subtypes for - (A) k = 6 and (B) k = 5, randomly picked from all available complete genome sequences

After confirming the efficacy of our method with word length of 6 with the Reference Sequence dataset, which comprised of homogenous representation of each subtype, we constructed the CGR of all available whole genome sequences from subtypes A to K (Test Set). We randomly picked up 45 sequences, with unequal representation of different subtypes, from the complete whole genome dataset and obtained the dendrogram with k = 6, as shown in Figure 5A. It is clear from Figure 5A that the Test sequences for HIV-1 subtypes segregated into different clusters with 100% accuracy - sequence 1 to 18 belonged to subtype B; 19 to 31 to subtype C; 32 to 36 to subtype D; 37 to 39 to subtype H; 40 to 42 to subtype J; 43 and 44 to subtype K and 45 was SIV_CPZ _sequence. We performed the same analysis to test whether taking lower word lengths can resolve the subtypes, and found that k = 5 gave incorrect clustering for one subtype C sequence (circled in Figure 5B). Thus, word length 6 can differentially segregate HIV-1 subtypes with both equal and unequal representation of subtype sequences. It may also be noted that the method was developed based on test sequences belonging to subtypes A, B, C, D, and G (Figure 3). Still it is able to identify and differentially cluster other subtypes, such as H, F, J and K, and sub-subtypes correctly.

### Predicting the subtypes of unclassified sequences

**Figure 6:**
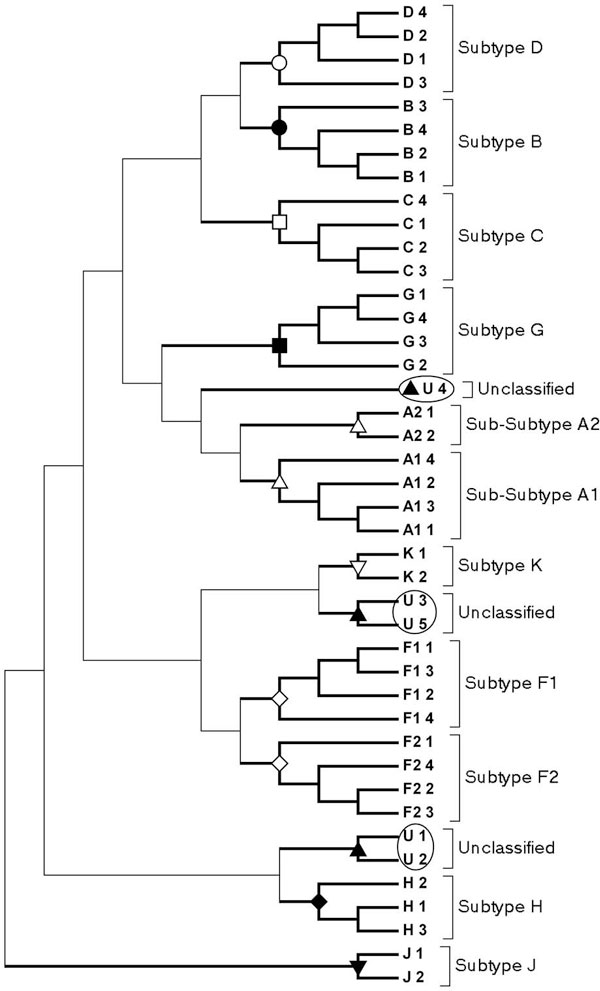
**Clustering of five Unclassified (U) sequences with the reference set of HIV-1 group M subtypes**.

Some of the HIV-1 group M sequences are regarded as *Unclassified *sequences (U). These sequences either represent a variant that may belong to a new subtype, or has been left unclassified due to lack of proper classification methodology. We constructed the CGRs from the whole genome sequences of the five Unclassified entries (U1-U5), and used the HIV-1 Reference Sequence set to cluster them. The dendrogram is shown in Figure 6. The two unclassified sequences (U1 and U2) are from Democratic Republic of the Congo, and were sequenced in year 1983 and 1990 respectively. They form a distinct cluster close to subtype H cluster. From the sub-subtypes clustering patterns in the dendrogram, we suggest that these sequences may be regarded as subtype H variants. In this respect, it may be noted that H subtype is also found in the Central Africa and is prevalent in Democratic Republic of Congo. The unclassified sequences U3 and U5, from the Netherlands, were sequenced in year 1995 and 2001, respectively. These two form a distinct cluster close to subtype K. Similar to U1-U2, these two (U3 and U5) can be regarded as subtype K variants. It may be mentioned that U3 and U5 have been shown to be similar to subtype K in certain limited regions of the sequence length of 100 to 200 nucleotides [28]. In that study, the authors pointed out that these sequences might be designated as subtype K variants, provided more sequences are obtained from unrelated individuals, as it was not commonly found in Netherlands [28]. Unclassified sequence U4, isolated from Greece, is closest to subtype A cluster, however, it does not cluster along with either of the A sub-subtypes. Our result indicate that this may be another A sub-subtype. Studies have shown that subtype A has become prevalent in Greece and these variants, though similar to subtype A, do not belong to any of the subtype A sub-clusters [29].

## Conclusion

The exponential growth of HIV-1 genome sequences manifest variable geographical distribution of the subtypes. Subtype C dominates the regions of South Africa, India, China, etc. and is the most prevalent subtype that causes HIV-1 epidemic. On the other hand, HIV-1 subtype B is the most studied subtype predominant in Americas, Australia, Western Europe, Japan etc. The high variability of HIV-1 has major influence on infectivity and transmissibility of the virus, with subtypes exhibiting variable treatment response and differential selection of drug resistance mutations as seen for subtype B and C [7]. Therefore, accurate classification of HIV-1 genomes is important as it facilitates the efficacy of monitoring the epidemic and developing treatment strategies.

CGR in the past has been used as a visualization tool, and it was shown that it could highlight inter-species differences. However, here we propose that CGR is a simple and computationally less intensive method, which can even identify genomic signatures marking intra-species variability, which are much less as compared to the inter-species variability. We demonstrate that CGR is a suitable method to correctly separate HIV-1 group M subtypes, using whole genome sequences. We demonstrate the applicability of different word lengths, and prove that word length of six is sufficient to differentially segregate HIV-1 subtypes and sub-subtypes into distinct clusters. In HIV-1, regions of high variability have been known to exhibit non-random distribution of certain 6 base pair long nucleotide sequences, which may undergo non-synonymous mutations leading to changes in the amino acids [30]. CGR computed from genome sequences, however, can recognize both synonymous and non-synonymous changes highlighting both neutral as well as selective mutations. It remains to be studied if a similar analysis of the proteome, instead of the genome sequence, would be useful in obtaining the functional basis of the sub-type classifications.

This methodology utilizes genome-wide information rather than gene- or region-specific information to classify HIV-1 subtypes. Using CGR, we could replicate the clustering of Reference Sequence set, and also all other HIV-1 group M subtype sequences. Importantly, we show that using this method we could also classify the five Unclassified sequences to subtypes, which fit with additional information available in literature. Thus, we demonstrate the applicability of this new method to solve the complex problem of HIV-1 sub-typing, and propose its use in subtype annotation of the newly sequenced HIV-1 genomes. The proposed methodology, with suitable word length optimisation, can also be applied to classify intra-species variants in other organisms.

## Competing interests

The authors declare that they have no competing interests.

## Authors' contributions

**AP: **Planned and carried out experiments, discussions and writing the manuscript.

**SS: **Plan of experiments, discussions, and writing the manuscript.

All authors read and approved the final manuscript.
